# The Chinese Mandarin Version of the Crisis Triage Rating Scale for Taiwanese with Mental Illness to Compulsory Hospitalization

**DOI:** 10.3390/ijerph182413392

**Published:** 2021-12-20

**Authors:** Shuo-Yen Ting, Tsuo-Hung Lan, Lih-Jong Shen, Chun-Yuan Lin, Shih-Kai Lee, Wei-Fen Ma

**Affiliations:** 1 Department of General Psychiatry, Tsaotun Psychiatric Center, Ministry of Health and Welfare, Nantou 54249, Taiwan; syting@ttpc.mohw.gov.tw (S.-Y.T.); thlan@nycu.edu.tw (T.-H.L.); cylin@ttpc.mohw.gov.tw (C.-Y.L.); 2 School of Nursing, Asia University, Taichung 41354, Taiwan; 3 Institute of Brain Science, National Yang Ming Chiao Tung University, Taipei 112304, Taiwan; 4 Department of Medicine, National Yang Ming Chiao Tung University, Taipei 112304, Taiwan; 5 Center for Neuropsychiatric Research, National Health Research Institutes, Miaoli 35053, Taiwan; 6 Institute of Clinical Medicine, National Yang Ming Chiao Tung University, Taipei 112304, Taiwan; 7 Department of Mental and Oral Health, Ministry of Health and Welfare, Taipei 115204, Taiwan; ljshen470507@gmail.com; 8 Department of Sport, National Changhua University of Education, Taichung 41354, Taiwan; 9 Department of Nursing, Tsaotun Psychiatric Center, Ministry of Health and Welfare, Nantou 54249, Taiwan; sklee@ttpc.mohw.gov.tw; 10 PhD Program for Health Science and Industry, China Medical University, Taichung 406040, Taiwan; 11 School of Nursing, China Medical University, Taichung 406040, Taiwan; 12 Department of Nursing, China Medical University Hospital, Taichung 404332, Taiwan

**Keywords:** psychiatry, instrument, compulsory hospitalization, Chinese Mandarin version of crisis triage rating scale

## Abstract

Background: A controversial issue of the need to protect human rights and ensure public safety still remains a conflict in Taiwan. The purpose of this study was to translate the Crisis Triage Rating Scale to Chinese Mandarin (CMCTRS). Method: A cross-sectional design with convenient sampling was employed in this study. The CMCTRS was tested on 302 Taiwanese individuals with mental illness who were admitted to the emergency room (ER) of a psychiatric center. A higher score indicated a greater need for mandatory psychiatric admission. Psychiatrists rated the patients’ status according to three scale criteria and six action plans of recommendations. Results: Five specialists evaluated the content validity index to be 0.8. A total of 210 participants (69.5%) were deemed suitable for compulsory hospitalization or admission for observation in ER. The optimal cut-off score was 8, with a Youden Index of 1.46, a sensitivity of 0.748, and a specificity of 0.712 in deciding the need for hospitalization or observation. Conclusions: The CMCTRS exhibited an acceptable criterion validity with psychiatrists in a population of 302 patients at the ER of a psychiatric center. A cut-off point of 8 is recommended for determining hospitalization or a minimum 24 h stay at emergency for observation.

## 1. Introduction

In Taiwan, 8.49 mental health beds per 10,000 people in Taiwan are sufficient to meet the need for the hospitalization of most who are mentally ill [1]. However, after being discharged from hospitals, some people who suffer from mental illness may remain unstable and be in need of medical assistance when they return to their communities [2]. Existing and incomplete strategies seldom detect problems in time for community-dwelling psychiatric patients. This has resulted in more than 5000 patients with confirmed and suspected mental illnesses who were sent to an emergency room (ER) every year [1], according to the data of Ministry of Health and Welfare of the Department of Mental and Oral Health. This poses a formidable challenge to emergency and medical professionals, as they have to decide whether these psychiatric patients are ill enough to merit compulsory hospitalization. More often than not, such decisions are not based exclusively on professional medical judgment, but police officers, paramedics, public health nurses, and social workers may all provide other views besides medical perspectives, especially legal and human viewpoints [3].

Article 15 on mandatory hospitalization in the Mental Health Act [4] stipulates that matters pertaining to mandatory hospitalization and community treatment due to mental illness shall be reviewed by the Mental Illness Mandatory Assessment and Community Treatment Review Committee. This committee advocates for patients’ rights and interests, as well as other relevant professionals [5]. Article 32 of the same act prescribes that police and/or fire-fighting agencies should inform local competent authorities when finding patients and/or persons who are about or likely to harm others and/or themselves. Police officers and fire fighters are required by law to escort the patients and/or persons to nearby medical care institutions for medical care unless specified otherwise [4]. However, the regulations provide only general guidelines and clear cause-and-effect relationships between the psychiatric disorders that individuals suffer and their harming themselves and/or others are often difficulty to establish in clinical settings [3].

Regulations related to mandatory hospitalization are usually too imprecise to provide clear, specific guidelines for establishing the possibilities and consequences of patients harming themselves and/or others [6]. Disagreements between specialist physicians that recommend compulsory psychiatric admission and other committee members occur frequently. Furthermore, legal experts have been identified as the group who voice disapproval the loudest [5]. Therefore, a standardized screening instrument with recognized credibility is imperative [7]. It should provide police officers and/or paramedics with a solid basis for taking psychiatric patients to an ER [8]. Frontline public health nurses and home-care teams [9] will also be able to employ the screening tool optimally to reduce the risk to community safety.

The Crisis Triage Rating Scale (CTRS) is a mental health triage scale developed by Bengelsdorf et al. in 1984 [10]. The CTRS is a three-tier scale that assesses an emergency psychiatric patient’s severity of mental illness. It has three subscales: dangerousness, which evaluates the danger that the patients pose to themselves and others; support system, which comprises family members, friends, and/or community resources that are capable of supporting and taking care of the patient; and the ability to cooperate, that is, patients’ ability to understand their own needs and work with others to meet those needs [10]. The score of each subscale ranges from 1 point to 5. The degree of recommended action or emergency is based on the total score of the three subscales [11].

The Statewide Mental Health Triage Scale [12] introduced by Victoria Government, Department of Health, Australia in 2010, has seven tiered triage codes but does not have the scoring system. Natisha et al. developed The UK Mental Health Triage scale in 2016 [13]. There are similar seven triage codes according to levels of urgency. However, the lack of quantization in these two triage scales may cause difficulty and inconsistency between triage raters. Hoffman et al. published interRAI Brief Mental Health Screener developed in Ontario Canada in 2016 [14]. It included 14 variables as good predictors of who was most likely to be taken to hospital by police officers and who was most likely to be admitted. There are no levels of urgency and recommended medical service response accordingly. These deficiencies making patients’ arrangement disposition difficult to decide.

A concordance rate as high as 97% was realized when the CTRS-based recommended actions were compared to those prescribed by clinical psychiatrists [10]. The CTRS has gradually become a popular choice as a mental health triage tool that is easy to operate. The CTRS was efficient in reaching reliable results because of its excellent criterion validity among medical professionals [10]. However, it cannot be employed for patients with alcohol intoxication and/or who are taking medication that may cause rapidly changing symptoms [10,11]. It has been suggested that nine points may be a good cut-off score to measure the relationship between the CTRS score and the decision whether to hospitalize the patient. While a score above nine indicates the need for compulsory hospitalization, a score below nine suggests outpatient crisis intervention treatment may be employed [10]. The action plans [12] based on the CTRS score are divided into seven levels, ranging from Level A, which necessitates an immediate response that requires the police or ambulance services and/or other services, to Level G, which implies that no further action is required after proper counsel. There are recommended actions with corresponding time frames for each level [15].

The CTRS had also been employed to test 500 individuals with mental illness who visited the ER in a hospital in Ontario, Canada. The results thereof indicated that a score of 9.32 points could serve as a good cut-off point to assess the need for hospitalization [16]. In addition, hospitalization was recommended for 61.2% of the assessed participants who scored eight points. This percentage escalated to 81.5% for those who scored nine points. If the optimal cut-off score for hospitalization was set at nine points in Turner, the rate of recommended hospitalization by CTRS would be 75.2%, which concurs with the clinical assessment. This would increase to 81.2% if the cut-off score was set at eight points. However, Turner set the cut-off for mandatory hospitalization at nine points after consideration of the correlation between the CTRS score and rate of recommended hospitalization.

Another study on 247 emergency psychiatric patients in a Nigerian hospital set the cut-off score at 10 points to distinguish emergent cases from urgent ones in accordance with a consideration of the sensitivity and specificity of the CTRS [11]. The Nigerian study [17] further compared the CTRS with the Clinical Global Impressions scale (GCI) and found the former had satisfactory criterion validity. Moreover, with accumulated empirical support, in 2012, the CTRS was proposed in a directive entitled Mental Health Triage Policy, which the Ministry of Health, NSW, Australia promulgated [15] as the standard for mental health telephone triage services offered by the state’s health organizations, community health centers, public hospitals, mental health clinical/patient services, and other affiliated medical institutions.

So as to address the need for a standard mental health triage scale to help ER professionals assess the need for hospitalization or observation of psychiatric patients in Taiwan, the original version of the CTRS and the guidelines for its recommended uses, which are provided in the Mental Health Triage Policy that the New South Wales (NSW) Ministry of Health announced in 2012, was consulted so as to translate the CTRS into Chinese Mandarin. The translated scale, named the Chinese Mandarin Version of the Crisis Triage Rating Scale (CMCTRS), was tested on actual emergency psychiatric patients in order to measure its validity and propose an optimal cut-off point adapted for Taiwanese patients.

## 2. Materials and Methods

### 2.1. Study Design and Study Patients

A cross-sectional design was used for this study to recruit patients with mental illnesses who had visited the ER of a psychiatric hospital in central Taiwan. The hospital, which operates a 24 h ER, has 193 beds and a further 800 for acute and chronic psychiatric patients, respectively. The participants included patients who had visited the ER of the hospital between July and December 2019. The ER had 30 senior psychiatrists and four psychiatric residents who all attended a meeting in which the structure and details of the CMCTRS, the different levels of recommended responses, the purpose of the study, participant recruitment and exclusion criteria, and other essential parameters of the study were explained. The senior and residents of emergency psychiatrists completed the rating of 210 and 154 triaged patients, respectively.

### 2.2. Instruments

Three major instruments were employed to collect data, namely, a demographic data sheet, the CMCTRS, and the Taiwan Urgency of Response Scale (TURS). While the content and wording of the CMCTRS is the same as the original CTRS, its scoring is the opposite. The TURS, which is based on the Urgency of Response Scale of the Mental Health Triage Policy [12,15], was modified slightly to meet the needs of Taiwanese users more appropriately.

#### 2.2.1. Demographic Data Sheet

A demographic data sheet was used to collect information on participants’ age, gender, marital status, and experience of staying in an acute psychiatric ward. The emergency psychiatrists completed this demographic sheet from the participants’ medical records within 24 h after the patients had been discharged from the ER.

#### 2.2.2. Chinese Mandarin Version of the Crisis Triage Rating Scale (CMCTRS)

The CMCTRS comprises three items that assess dangerousness, support system, and ability to cooperate. Scores range between 3 and 15 points [10]. The CTRS has been used in different regions and is known for its efficiency and effectiveness [10,16,17]. The scale was first translated from English into Chinese by the authors of the study. Subsequently, the translated version was back-translated from Chinese to English by a bilingual public health Ph.D. candidate who had lived in both Taiwan and Canada for over 15 years. The original English version and the translated version were then reviewed by two professors who specialized in semantics. While one was a native English speaker, the other was well versed in both English and Chinese. The translated version obtained a content validity index (CVI) of 1.0 that testified to the accuracy of the translation.

Subsequently, five psychiatry professors and senior mental health clinicians assessed the face validity and content validity of the CMCTRS in this study. The content of each item was evaluated to assess its compatibility with Taiwanese culture. A fairly acceptable CVI of 0.8 was obtained. Moreover, the specialists reached a consensus to change the scoring approach of the original CTRS, in which a higher score suggested a lower need for hospitalization, to one adopted for the CMSTRS, in which a higher score indicated a greater need for hospitalization, so as to ensure consistency with the scoring method with which most Taiwanese users of Chinese screening instruments are familiar. The intraclass correlation coefficient was 0.75 for 28 patients rated by two different psychiatrists. Meanwhile, the Kappa was 0.63 for dangerous items, 0.54 for support system items, and 0.70 for cooperate items in this study.

#### 2.2.3. Taiwan Urgency of Response Scale (TURS)

The TURS comprises of only one item that was modified from the Urgency of Response Scale of the Mental Health Triage Policy [12]. The item requires the responsible psychiatrist to indicate the recommended response or action plan based on their clinical assessment and experience.

The Mental Health Triage Policy promulgated by the Ministry of Health in 2012 and the Triage Scale [12]: Mental Health Service Guidelines published by the Department of Health, Victoria, Australia, were employed to acquire a more in-depth understanding of how the CTRS reflects different levels of need, risk, and urgency. The mental health resources that are currently available and corresponding emergency responses that are actually affordable in Taiwan were then considered. To meet the localized situations in Taiwan appropriately, the CMSTRS encompasses the original seven (A to G) categories of urgency or levels of response that were condensed into the following six categories/levels to establish a TURS: acute care hospitalization, ER admission for observation, next-day outpatient appointment or home visit, outpatient appointment or home visit in three days, outpatient appointment or home visit in two weeks, and others such as referral to a nonmedical institution and no follow-up necessary. The agreement of TURS examined by Kappa was 0.46 (*p* < 0.05) in this study.

### 2.3. Study Procedure and Ethical Considerations

The approval was obtained from the institutional review board (IRB) (NO. 108007) to protect the participants’ rights. The research involved no more than minimal risk to study participants. The explanation of study purpose, procedure, and verbal consent was obtained from emergency psychiatrists before data collection. Patients were examined and received treatment from the emergency psychiatrists. Subsequently, the three instruments were reviewed and completed by the emergency psychiatrists in accordance with their clinical judgment. Moreover, whether the patients had completed the scale did not affect their medical treatment and the service to which they were entitled. The decision to admit a patient was based exclusively on a clinical risk assessment, which the responsible psychiatrist conducted, as well as the capacity of the relevant support system. Whether the hospital had a bed available, whether there was any other external factor that might prevent hospitalization, and previous arrangements between the patient and the hospital and its medical professionals were not taken into consideration.

### 2.4. Study Analysis

Descriptive and inferential statistics were utilized to analyze the participants’ demographic data. In accordance with the participants’ CMCTRS scores and the TURS responses the emergency psychiatrists had rated after examining the participants’ mental states and analyzing their medical history, optimal cut-off points were determined. Logistical regression analysis was performed to measure the sensitivity and specificity of the cut-off score of CMCTRS for hospitalization or admission for observation. The Youden Index [18] and receiver operating characteristic (ROC) curve analysis were employed to determine the cut-off scores.

## 3. Results

### 3.1. Demographic Data

Of the 364 participants that were recruited, all the instruments were completed for 302. The mean age of the 302 participants was 45.9 years (SD = 15.7). Females (*n* = 171, 56.6%) and those aged between 45 and 54 years (*n* = 82, 27.2%) formed the largest group of participants. Furthermore, 63.6% (*n* = 191) were single and 71.9% (*n* = 217) had been treated in an acute ward. After completing the CMCTRS, acute care hospitalization and admission for observation were recommended for 210 participants (69.5%), of which 202 (66.9%) required acute care hospitalization.

### 3.2. The CMCTRS

The participants’ average CMCTRS score was 8.38 points (SD = 2.47), with the 50th percentile (the median) falling within 8.0 points. The average scores for the dangerousness, support system, and ability to cooperate subscales were 3.0 points (SD = 1.39), 2.36 points (SD = 1.25), and 3.02 points (SD = 1.17), respectively. Furthermore, while 19.5% of the participants were given the worst rating (5 points) for dangerousness, 3.6% and 13.6% were rated 5 for ability to cooperate and support system, respectively (Table 1).

### 3.3. The TURS

Of the 302 participants, 202 (66.9%) required acute care hospitalization, 8 (2.7%) ER admission for observation, 7 (2.3%) next-day outpatient appointment or home visits, 38 (12.6%) outpatient appointment or home visit in three days, 26 (8.6%) outpatient appointment or home visit in two weeks, and 21 (7.0%) others, including referral to a non-medical institution and no follow-up necessary. Because the purpose of the study was to assess the need for further intervention among community-dwelling psychiatric patients sent to ER, while those recommended for acute care hospitalization and ER admission for observation were placed into one category, namely, hospitalization (*n* = 210, 69.5%), the other four recommended responses were classified as other option (*n* = 92, 30.5%).

Thus, there were two major action plans. No significant differences were observed between the participants in the two categories in relation to age, sex, and marital status. The only statistically significant difference (*ꭕ*^2^ = 19.89, *p <* 0.000) was the experience of staying in an acute psychiatric ward (Table 2). Furthermore, 77.4% of the participants with experiences of staying in an acute psychiatric ward were recommended for acute care hospitalization or ER admission for observation. This was higher than that of the participants with no previous experience of staying in an acute ward (49.4%).

### 3.4. The Cut-Off Point Analysis of CMCTRS

The participants for whom hospitalization was recommended had an average CMCTRS score of 9.2 points, with the median falling on 9.0 points. The average CMCTRS score of all the other participants was 6.5 points, with the median falling on 6.0 points. While those for whom hospitalization was recommended scored an average 3.3 points in the dangerousness subscale, this was significantly higher than their counterparts who scored 2.2 points. Hospitalization was recommended for approximately 85.7% of the participants who scored over 4 points in the dangerousness subscale (Table 3).

Those for whom hospitalization was recommended had an average score of 2.6 points for the support system subscale, which was significantly higher than the 1.9 points of their counterparts. Moreover, hospitalization was recommended for as many as 88.2% of the participants who scored over 4 points in the support system subscale. In relation to ability to cooperate, while the average score of those for whom hospitalization was recommended was 3.3 points, their counterparts scored 2.3 points for this subscale. Approximately 80.0% of the participants who scored over 4 points in this subscale were recommended for hospitalization.

**Figure 1:**
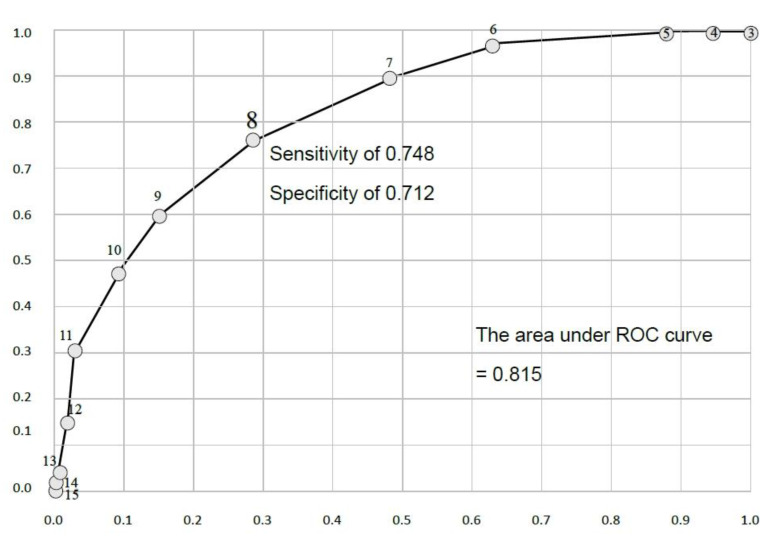
ROC curve analysis to determine the cut-off scores of CMCTRS.

The results revealed the maximum value of the Youden Index was 8 (Table 4) and further indicated the optimum CMCTRS cut-off point was 8, and sensitivity and specificity 0.762 and 0.717, respectively. The area under ROC curve was 0.815 (cut-point = 8) (Figure 1). In essence, a CMCTRS score of 8 points or higher suggests hospitalization is required.

## 4. Discussion

### 4.1. Main Findings

The CMCTRS, which was the result of the first attempt to translate the original CTRS into Chinese, was tested and demonstrated good face validity and content validity as well as a satisfactory equivalence with the original English version. More importantly, the CMCTRS was modified in response to recommendations of specialists to localize its criteria in a manner to ensure it is more capable of identifying psychiatric patients with a higher total score, thus indicating a more severe condition and poorer support system for interventions and responses actually available in Taiwan. This particular feature of localization renders the CMCTRS an effective and efficient screening tool that clinical psychiatrists can employ to decide on appropriate responses, including acute care hospitalization and admission for the observation of individuals with mental illness who visit an ER in Taiwan.

The results of this study set the optimal CMCTRS cut-off point at eight points, at which the best sensitivity and specificity with the Youden Index was noted [18], with a maximum value of 1.479 points and an ROC area of 0.815. A patient with a mental illness who scores eight or more points on the CMCTRS should be compelled to either go to hospital or be admitted for observation in an ER. Therefore, a CMCTRS score of eight points can be considered to be an essential criterion for clinical psychiatrists to judge whether an individual with a mental illness who visits an ER should be admitted to hospital for acute care or psychiatric observation, or alternatively, undergo a community-based crisis intervention.

Bengelsdorf [10] revealed that 147 (49%) of 300 subjects who were receiving crisis triage were hospitalized with an average CTRS score of 11.8 points. In our study, 66.9% of the assessed participants for whom hospitalization was recommended had an average CMCTRS score of 9.2 points. It is noteworthy that the host hospital in Bengelsdorf [10] was the only institution authorized to compel psychiatric patients in the region to be admitted, thus implying that patients sent to the host hospital were more likely to exhibit a higher degree of dangerousness. Bengelsdorf [10] noted that the host hospital had made continuous efforts to refine its crisis triage criteria so as to enhance its decision of patients requiring hospitalization or non-hospitalization options [11]. This may help explain why a more rigorous cut-off score of nine points was recommended. It is also noteworthy that in Bengelsdorf [10], the host hospital had a crisis response team that could readily be mobilized to meet the needs of community interventions at any time. Bengelsdorf recommended that the CTRS cut-off score be lowered to eight or even seven points [10] in regions where mandatory hospitalization was difficult to implement or no crisis response service was available [11]. This is the current status of psychiatric care in Taiwan, where compulsory hospitalization needs to meet rigorous criteria and 24 h crisis response teams are lacking. The optimal cut-off score for mandatory hospitalization at eight points in this study appears to be consistent with Bengelsdorf’s recommendation and compatible with the actual status of psychiatric care in Taiwan. Therefore, the CMCTRS can be expected to serve as acceptable guidance to assist clinical psychiatrists in emergency psychiatric evaluation.

Moreover, in contrast to the CTRS cut-off score of nine points, in this study, the CMCTRS cut-off score was set at eight points to accommodate cultural differences between Taiwan and Western countries. In Australia, Canada, and the United States, deinstitutionalization is at the core of public mental health policies. Furthermore, community interventions for promoting mental health and protecting psychiatric patients are better established [19]. Meanwhile, deinstitutionalization has resulted in a drastic reduction in the number of psychiatric beds, and it takes psychiatric patients three times longer to secure a bed than their non-psychiatric counterparts [20]. This may be a plausible reason for the more rigorous CTRS cut-off score of nine points, one point higher than the CMCTRS cut-off score of eight points.

The Convention on the Rights of Persons with Disabilities by the United Nations has entered into force since 2008 [21]. The convention reaffirms that all persons with all types of disabilities must enjoy all human rights and fundamental freedoms. The police officers’ involuntary escort services for psychiatric treatment have been under more surveillance by the government and the general public. They are under more pressure to deal with psychiatric emergency and feel most stressed by judging patient from non-patient, and deciding whether to escort patients to hospitals or not [22]. The trade-off between psychiatric patients’ human rights and public safety are becoming decisions more difficult to make [23].

Mental health triage tools assessing the level of dangerousness and risk of self-harm or harming others might be more objective and communicable platforms between law and psychiatry. Triage scales usually have different levels of urgency and recommendation for appropriate responses, according to the urgency [12,13,24,25]. The advantages of these triage scales are clear-cut response types and response time-frame after assigning levels of urgency to patients. The shortcoming is that determining levels of urgency is not easy by merely applying a qualitative description of typical presentations in each level. In addition, the lack of quantifiable information also impedes but not facilitates more efficient handover in collaborative team work during psychiatric emergencies in the community.

The results also revealed a linear correlation between the scores in the dangerousness and ability to cooperate subscales and the recommended response of compulsory hospitalization or admission for observation. The participants who scored higher in the two subscales appeared to be more likely to be recommended for hospitalization or admission for observation. This correlation concurs with the general logic of clinical decision making in several previous studies [26]. However, compulsory hospitalization or admission was recommended for 72.7% and 88.2% of the participants who scored five points and four points, respectively, in the support system subscale. This may be explained by the difficulty of performing an accurate assessment of a patient’s family and/or social support systems in an ER setting [27]. The support system may be absent, which may cause the patient to feel agitated, act aggressively, and/or remain wary of the emergency psychiatrist [26,27].

### 4.2. Limitations and Recommendations

Despite its acceptable validity [26] in helping emergency psychiatrists rate the severity of patients’ mental illness and recommend appropriate responses, the CMCTRS is limited in its consideration of issues related to patients’ human rights [28]. To ensure psychiatric patients visiting an ER pose no threat to themselves and/or others, whether the dangerousness subscale should be employed as an independent criterion [29] to facilitate considerations from the perspectives of clinical psychiatry, community safety, social factors, and human rights protection may require further studies.

Emergency psychiatrists were invited to use the newly translated CMCTRS to assess people with mental illness who were visiting an ER [30]. While the CMCTRS has emerged as a valid screening tool, it should not replace the assessment of other medical and healthcare professionals involved in mental care. The symptoms and behaviors of a person with mental illness in an ER setting may differ considerably from those in a community setting. Therefore, the CMCTRS should be employed to assess only psychiatric patients in an ER. Its cut-off score of eight points may not be relevant in a community setting where judgment on optimal responses and/or interventions requires that the opinions of paramedics, police officers, public health professionals, and even social workers should be considered.

Most of the dangerousness to oneself or others resulted from psychotic symptoms, such as delusion, hallucination, or irritability [14]. To accurately and comprehensively examine psychopathology greatly relies on training and clinical experiences in psychiatric settings in hospitals. We would recommend limiting the usage of the instrument to those healthcare professionals with current or past experience of psychiatric acute services. Besides, we have noted the scoring point three and four are the most difficult to differentiate when applying the CMCTRS to real-world patients during the study. In order not to blur the line when making importance decisions, the accurate scoring ability is the fundamentally necessary competency of the triage raters [31]. It will be more helpful for the trainees to master the scoring by case scenario practice and case conference discussion on continuous education. Training workshops and certificates issued for those who were qualified in using the instrument might be suggested to protect both patients’ safety and human rights.

It should be further noted in this study that the CMCTRS was examined as an assessment instrument. The adequacy of the original three subscales, and, in particular, the support system subscale was not explored, and accordingly, a more effective replacement was not considered. It is recommended that future studies should expand their scope to include the experiences and opinions of frontline paramedics, caregivers, and even patients themselves so as to identify additional [13], more significant subscales to enhance the CMCTRS’ screening capacity.

## 5. Conclusions

After the translation and back-translation process, the CVI of 0.8 was evaluated by five specialists to ensure that the semantics and culture of the CMCTRS was localized sufficiently for Taiwanese people. The 302 enrolled patients had an average score of 8.4 points (SD = 2.57). Among them, 210 (69.5%) patients were deemed suitable for compulsory hospitalization or admission for observation. The optimal cut-off score was eight, with a Youden Index of 1.46 and an area of the ROC curve of 0.80. The scale had a sensitivity and specificity of 0.748 and 0.712, respectively, in screening emergency psychiatric patients to decide on the need for hospitalization or observation. The CMCTRS, which is based on the CTRS, exhibited an acceptable criterion validity with psychiatrists in a population of 302 patients at the ER of a psychiatric center. A cut-off point of eight is recommended for determining hospitalization or a minimum 24 h stay at an ER for observation.

## Figures and Tables

**Table 1:** Number of Participants (%) by CMCTRS Scores (*n* = 302).

Score	Rating A: Dangerousness	Rating C: Support System	Rating B: Ability to Cooperate
1	53 (17.6%)	39 (12.9%)	95 (31.5%)
2	74 (24.5%)	43 (14.2%)	93 (30.8%)
3	53 (17.6%)	134 (44.4%)	27 (8.9%)
4	63 (20.9%)	45 (14.9%)	76 (25.2%)
5	59 (19.5%)	41 (13.6%)	11 (3.6%)

Rating A, 1 = less dangerousness to 5 = greater dangerousness. Rating B, 1 = higher support system to 5 = poor support system. Rating C, 1 = more cooperation to 5 = lower cooperation.

**Table 2:** Demographic data analysis in TURS recommended response.

Variable	Other-Option	Hospitalization	*p*-Value
All	44.9 (17.1)	46.3 (15.1)	0.4592
Age Range			
12–24.9	10 (31.3%)	22 (68.8%)	0.0824
25–34.9	19 (46.3%)	22 (53.7%)	
35–44.9	19 (27.5%)	50 (72.5%)	
45–54.9	17 (20.7%)	65 (79.3%)	
55–64.9	17 (37.0%)	29 (63.0%)	
Over 65	10 (31.3%)	22 (68.8%)	
Gender			
Male	47 (35.9%)	84 (64.1%)	0.0736
Female	45 (26.3%)	126 (73.7%)	
Marital Status			
Married	23 (33.8%)	45 (66.2%)	0.6802
Single	56 (29.3%)	135 (70.7%)	
Widowed	5 (41.7%)	7 (58.3%)	
Divorced	8 (25.8%)	23 (74.2%)	
Experience of Staying at an Acute Psychiatric Ward			
Yes	43 (50.6%)	42 (49.4%)	<0.0001
No	49 (22.6%)	168 (77.4%)	

Number of participants (%); *p* value of chi-square test.

**Table 3:** Scores of hospitalization and other option participants.

CMCTRS Score	Hospitalization (*n* = 210)	Other-Option (*n* = 92)	*p*-Value
Rating A: Dangerousness	3.3 (1.3)	2.2 (1.2)	<0.0001
1 (Less)	24 (45.3%)	29 (54.7%)	
2	41 (55.4%)	33 (44.6%)	
3	38 (71.7%)	15 (28.3%)	
4	54 (85.7%)	9 (14.3%)	
5 (Greater)	53 (89.8%)	6 (10.2%)	
Rating B: Support System	2.6 (1.3)	1.9 (1.1)	<0.0001
1 (Higher)	55 (57.9%)	40 (42.1%)	
2	60 (64.5%)	33 (35.5%)	
3	20 (74.1%)	7 (25.9%)	
4	67 (88.2%)	9 (11.8%)	
5 (Poor)	8 (72.7%)	3 (27.3%)	
Rating B: Ability to Cooperate	3.3 (1.1)	2.3 (1.1)	<0.0001
1 (More)	13 (33.3%)	26 (66.7%)	
2	19 (44.2%)	24 (55.8%)	
3	104 (77.6%)	30 (22.4%)	
4	36 (80.0%)	9 (20.0%)	
5 (Lower)	38 (92.7%)	3 (7.3%)	

Mean differences analyzed by *t*-test.

**Table 4:** Sensitivity, specificity, and Youden Index of CMCTRS score for determining hospitalization/admission.

CMCTRS Score	Sensitivity	Specificity	Youden Index
3	1.000	0.000	1.000
4	1.000	0.054	1.054
5	0.990	0.130	1.121
6	0.962	0.370	1.331
7	0.890	0.522	1.412
8	0.762	0.717	1.479
9	0.595	0.848	1.443
10	0.471	0.913	1.384
11	0.314	0.967	1.282
12	0.157	0.978	1.135
13	0.062	0.989	1.051
14	0.033	1.000	1.033
15	0.005	1.000	1.005

Youden Index = sensitivity + specificity.

## Data Availability

These study data are identified participant data. The data that support the findings of this study are available beginning 12 months and ending 36 months following the article publication from the corresponding author, W.-F.M., upon reasonable request at lhdaisy@mail.cmu.edu.tw.
